# In Support of Meaningful Assessment and Feedback: A Study of Clinical Reasoning Tasks Used in Ambulatory Case Reviews

**DOI:** 10.5334/pme.2294

**Published:** 2026-03-13

**Authors:** Jacqueline M. I. Torti, Susan Humphrey Murto, Kristen A. Bishop, Azin Ahrari, Mark Goldszmidt

**Affiliations:** 1 Department of Medicine, Schulich School of Medicine and Dentistry, Western University, London, ON, Canada; 2 Centre for Education Research and Innovation, Schulich School of Medicine and Dentistry, Western University, London, ON, Canada; 3 Department of Medicine and Department of Innovation in Medical Education, Faculty of Medicine, University of Ottawa, Ottawa, ON, Canada; 4 Centre for Education Research & Innovation, Department of Medicine, Schulich School of Medicine and Dentistry, Western University, London, ON, Canada; 5 Department of Medicine and Division of Rheumatology, University of British Columbia, Vancouver, BC, Canada; 6 Division of General Internal Medicine, Department of Medicine, Schulich School of Medicine and Dentistry, Western University, London, ON, Canada

## Abstract

**Introduction:**

Assessing clinical reasoning and offering meaningful feedback to residents through workplace-based assessments remains challenging. Part of this challenge lies not only in articulating how well residents perform clinical tasks, but also in having a shared language to describe the underlying reasoning that supports those tasks. This study aimed to explore clinical reasoning tasks in an ambulatory setting and their relationship to clinical expertise.

**Method:**

This single-site instrumental case study explored clinical reasoning of junior and senior residents in new and follow-up rheumatology cases. Case reviews, including resident, attending, and patient interactions were audio-recorded and transcribed. Data analysis combined template and content analysis to explore reasoning. Coding was iterative, with regular consensus meetings among researchers. Findings were confirmed through a focus group with clinic faculty and residents.

**Results:**

53 cases were reviewed in ambulatory rheumatology clinics. New consultations focused more on tasks of identifying most likely diagnosis and establishing management plans, whereas follow-up encounters focused on assessing rate of progression, response to treatment, estimating prognosis, and determining follow-up. There were no consistent differences in the number of tasks addressed between junior and senior residents, nor between strong and weak case presentations. However, strong presentations demonstrated selectivity in addressing tasks related to specific patient needs.

**Discussion:**

Sharing common patterns of reasoning tasks addressed and omitted can better prepare residents for ambulatory encounters. A shared language around metacognitive tasks can enhance feedback and guide assessment instruments in competency-based training, helping residents broaden their perspectives beyond individual cases.

## Introduction

Fundamental to workplace-based assessment in medicine is the capacity to both judge a resident’s stage of competence and articulate specific, actionable priorities for growth to progress, both of which can be challenging to achieve in practice [1,2,3,4]. In part, the challenge relates to being able to not only assess how well they perform clinical tasks but the underlying reasoning that shapes these tasks. Assessing clinical reasoning in the workplace is inherently difficult [5,6], in part because assessor cognition, context and the tacit nature of reasoning complicate observation and feedback [1,7]. One promising approach involves developing an understanding of what residents are reasoning about during their encounters (i.e., the specific clinical reasoning tasks they address) [8,9,10]. Approaching workplace-based assessment through a reasoning task lens, may support better assessments and explicit, actionable feedback [6].

Although broadly understood, there is no single standard definition of clinical reasoning. Gruppen (2017) urges scholars engaging in work in this space to be explicit about the definition of clinical reasoning they are operationalizing, while being attuned to the inherent complexity of the term [5]. Accordingly, for the purposes of our work, we are operationalizing Gruppen’s (2017) description of clinical reasoning as a process through which clinicians synthesize their biomedical and clinical

knowledge with initial patient information to form a case representation of the problem. The physician uses this problem representation to guide the acquisition of additional information and then, on the basis of this information, revises the problem representation. [They] repeat the information gathering – representation revision cycle until [they] reach a threshold of confidence in that representation to support a final diagnosis and/or management actions [5].

This description encompasses both diagnostic reasoning and management (therapeutic) reasoning – the latter involves decisions about treatment, monitoring and follow-up, additional testing, shared decision-making, and resource allocation, and is comparatively underdeveloped in the literature [9,11,12]. While the development of clinical reasoning skills has been well described from a cognitive psychology perspective (e.g., progression from hypothetico-deductive reasoning to the use of illness scripts and pattern recognition) [6,13], practical application of these concepts to enable assessment and feedback in the workplace eludes many clinical teachers. Moreover, reasoning performance is context-specific and situated (i.e., shaped by patient, clinician, team, and system factors) [7,14] which is especially salient in ambulatory care. Prior work has begun to make explicit what physicians’ reason about (reasoning tasks) and to document how tasks vary with context, which is essential for considering developmental trajectories in real-world settings [8,9,10,15].

To date, teaching and assessing clinical reasoning tasks have largely been taken up in undergraduate education and in non-clinical settings, such as simulation or embedded in the classroom through problem-based learning [16,17,18]. And, while clinical reasoning tasks offer an explicit language for identifying and describing the underlying tasks to be addressed during a clinical encounter [8], they are not yet widely used to guide competency assessment in the clinical setting. Whether they can has also not been fully explored.

Although not always explicitly referred to as clinical reasoning tasks, prior studies have explored the link between reasoning tasks and clinical tasks to the hypothesis-driven physical exam [19,20], patient contextual features [21,22,23], and chronic active issues addressed in the clinical encounter [24,25]. While these studies have hinted at expertise effects, most were limited in scope, examining only a few reasoning tasks, and did not fully integrate an understanding of competence progression in clinical practice. However, they support the idea that competent practice involves nuanced reasoning tasks that guide clinical tasks.

The practical relevance of reasoning tasks during patient-physician interactions and their implications for competency and expertise development merits further study [9], particularly in ambulatory care. A prior study identified 24 reasoning tasks physicians may engage in during clinical encounters [8]. Subsequent validation in hospital admission case review showed these tasks’ utility in identifying fully, partially, or unaddressed tasks [10]. On average, teams attended to 17 of 24 reasoning tasks per admitted patient [10]. Three overarching tasks were identified – 1) *Identify Active Issues*; 2) *Determine Most Likely Diagnosis with Underlying Cause(s)*; and 3) *Establish Management Plan*. Overarching tasks were broader in nature and helped to organize some of the more specific reasoning tasks. In contrast, the remaining tasks were found to be context-specific and supportive of the overarching tasks [10]. Expertise was linked to addressing particular supportive tasks, like establishing goals of care, a reasoning task that residents were less likely to attend to [10].

How reasoning tasks are enacted and refined is shaped by context and should be studied within real-world clinical settings [7]. Yet, despite the predominance of patient care in ambulatory settings, research in the real-world context remains underexplored. Research in this area can build on existing work in inpatient settings [10] and further our understanding of how clinical expertise is developed, and provide key insights into how faculty can optimize targeted feedback for trainees [6,26]; ultimately improving patient care [6]. Rheumatology care occurs primarily in an ambulatory context and, thus, provides an excellent clinical setting to explore reasoning tasks further.

In sum, much of the clinical reasoning literature adopts socio-cognitive perspectives, is often classroom-based with undergraduate students, and has historically emphasized diagnosis. By contrast, management reasoning remains comparatively underexamined [9,11,12] especially in context-embedded ambulatory care. It also has been challenging for clinicians to adapt the knowledge into pragmatic skills/terms to inform workplace-based assessment and feedback [3,26], which is now central to competency-based medical education [3]. Examining clinical reasoning in action, in an authentic work-place setting, may offer a practical and actionable lens for assessment by providing a language for recognizing and describing the key elements that physicians can reason about during an encounter.

This study aimed to explore the types of clinical reasoning tasks (hereafter “reasoning tasks”) that residents engage in during ambulatory rheumatology care and the relationship between these tasks and clinical expertise. Ambulatory encounters include both new consultations and follow-up visits, each potentially requiring different reasoning tasks worthy of exploration. The specific objectives of this research were to explore:

1. Which reasoning tasks residents attend to during ambulatory case review, as a way to understand how clinical expertise is enacted and developed in this context.
  - What are the patterns of reasoning tasks that residents fail to address (i.e., omissions)?
  - How do these patterns of reasoning tasks differ as residents gain experience (i.e., junior vs. senior residents)?
2. How patterns of reasoning tasks differ between new patient consultations and follow-up encounters, to explore how visit type may shape reasoning task utilization.

## Methods

### Study Design

This single within-site instrumental case study [27], was bounded by the rheumatology ambulatory practice. The rationale for using a case study approach was threefold. First, case study designs are particularly well-suited to answering “how” questions – such as ‘how junior and senior residents differ in the reasoning tasks they attend to’ and ‘how these tasks differ between new patient consultations and follow-up encounters’ – because they support an in-depth, process oriented understanding of complex real-world phenomenon [28]. Second, case study designs allow for the observation of real-world phenomena through a naturalistic approach, enabling researchers to study events as they naturally unfold, without manipulating the behaviours of participants [28]. Third, case study designs are ideal for exploring contextual conditions relevant to understanding the phenomenon [28], essential for understanding reasoning tasks in the context of case reviews and thereby answering the call for more in situ research related to clinical reasoning [7]. In keeping with a case study approach, each case review was treated as a bounded “case” capturing in-context interactions between residents and attendings during routine ambulatory care. All case review conversations were audio-recorded, providing a naturalistic window into reasoning tasks as they unfolded in real time, consistent with the intent of case study designs to explore phenomena within their real-life context.

### Setting

This study was conducted in a rheumatology clinic at a Canadian academic health sciences center in a midsized urban area (catchment population 1 million). As a teaching center, residents see patients independently before presenting their findings to the attending physician, either outside of the exam room or sometimes inside the room (with the patient present). Patients in the clinic were referred for diagnosis and management of rheumatologic diseases. Rheumatology was chosen due to its focus on ambulatory care, a mix of new and follow-up cases, the complexity of disease management, and the involvement of both junior and senior residents.

### Participant Recruitment

Recruitment occurred in multiple stages. Attending physicians served as the starting point and were first invited via email, followed by phone calls if needed. Attendings were eligible for inclusion in this study if they were part of the academic ambulatory rheumatology clinic. Once attendings consented, residents who were scheduled to work with them during planned data collection times were invited to participate. Case reviews between residents and attendings were audio-recorded outside the patient room. In some cases, patient interactions were also recorded with patient consent, capturing the interaction between the resident, attending, and patient. Consistent with our case study approach, some attending physicians and residents contributed to multiple case reviews.

In alignment with a case study design, purposive sampling was used to inform data collection at two levels [28]. First, at the level of the resident, we sampled residents from the postgraduate years (PGY) 1–6 who participated in case reviews with attendings. This distinction allowed us to explore how reasoning task utilization may differ based on the resident’s stage of training. PGY 1–3 residents (currently in core internal medicine) in the program were considered junior for this study because they had limited ambulatory exposure in rheumatology as a subspecialty, whereas PGY 4–6 (currently in a rheumatology training program following three years of core internal medicine) were considered senior. Second, at the encounter level, sampling occurred based on case type, including both new consultations and follow-up cases. This approach was intended to capture variation in cases, which may influence the nature of case reviews and the types of reasoning tasks addressed.

### Data Collection

With consent from attending physicians, residents, and, when applicable, patients, a research assistant attended half-day clinics over a 10-month period to audio-record case review discussions. Following usual practice, residents first assessed patients independently, then stepped out to discuss their findings with the attending physician. These conversations outside the exam room were recorded, and in some cases, recording continued when the discussion moved into the examination room with the patient present.

Cases were selected based on the existing clinic schedule, with the intention of purposefully sampling at both the resident and encounter levels, to reflect variation in cases. The resulting dataset consisted of audio-recorded case reviews (both new and follow-up) between attending physicians and their residents, with some recordings also including conversations with patients who had provided consent. Several faculty and residents participated in more than one case review, offering insight into patterns of reasoning across varying clinical encounters.

### Data Analysis

Data collection and analysis occurred concurrently in an iterative fashion. A combination of template and content analysis was used to analyze individual case reviews [29,30]. A template analysis was developed using an existing framework of previously validated reasoning tasks [8,29], which served as a deductive coding guide (i.e., template) to identify the presence or absence of reasoning tasks within and across case reviews. The framework is outlined in Column 1 of Table 2 introduced in the results section below. This approach was used to address Objective 1 and 1a by identifying the reasoning tasks residents attended to and the omissions that occurred during case review. A constant comparative approach [31], supported comparisons across junior and senior residents (Objective 1b) and across new and follow-up encounters (Objective 2).

During the original coding session, the transcript was coded as it was read and then re-read and re-coded to ensure that later text data from the encounter informed the coding of earlier portions; this was necessary as some of the reasoning tasks were addressed in more implicit and subtle ways (e.g., in reading and coding the resident case summary in full, earlier comments became more clearly related to a particular reasoning task). The coding of future encounters also informed the re-coding of prior encounters.

We also conducted a qualitative content analysis [32] to address Objectives 1a and 2 by examining how attendings explicitly or implicitly signaled the importance of specific reasoning tasks, elaborated on certain tasks, and introduced tasks not addressed by the resident. This further analysis helped us to deepen our understanding of those tasks that are commonly addressed or unaddressed by residents.

Transcripts were independently and recursively coded by three researchers [JT, AA, SH], with regular meetings to discuss reasoning tasks and case review context. We engaged in reflexive conversations about how AA and SH’s professional experiences with case reviews shaped their interpretations. We also used memoing during analysis meetings to capture these reflections and how they may have influenced our coding decisions. Consensus coding was captured using NVivo software. Monthly meetings with two additional researchers [MG, KAB] addressed unclear reasoning tasks or tasks for which a consensus could not be reached. Discrepancies often involved recognizing reasoning tasks and their explicit or implicit nature. Prior to coding the full dataset, the team conducted training sessions to calibrate coding using a small subsample of transcripts. In these sessions, coders discussed their interpretation of the coding framework and resolved differences in understanding. This process supported the development of shared expertise in applying the framework. Once consensus in coding was achieved, coding switched from two coders to a single coder/case. In total, 30% of the case review transcripts had two independent coders, each with at least one clinician researcher analyst, and the remaining 70% of case review transcripts were coded by a single, independent coder with clinical expertise in rheumatology.

To enhance the trustworthiness and credibility of our findings, we conducted a return-of-findings focus group as a form of member-checking during rheumatology rounds to explore the resonance of our preliminary findings. An email was sent to all individuals on the grand rounds’ distribution list, inviting them to attend a return-of-findings focus group to learn about the study’s preliminary findings and share their feedback. The focus group was attended by 6 rheumatologists and 24 residents (some who were participants in the study and some who were not). Following informed consent from all participants, the session was audio-recorded and transcribed verbatim. The focus group was conducted by JT, AA, and MG with guiding questions aimed to clarify and refine key concepts, explore whether the findings resonated with attendees, and identify any missing elements. We shared our preliminary findings organized into a brief presentation on the reasoning tasks addressed in new admissions, follow-up cases, and those most commonly addressed or added by the attending. Following each brief presentation of a preliminary finding we asked, “Do these findings resonate with you?”; and “Is there anything that we missed?”. Focus group transcripts were inductively analyzed and served as a form of member checking to assess and refine our preliminary findings. The discussion revealed the importance of (in addition to audio-recording case-reviews between resident and attending) also audio-recording case reviews held between patients, attendings, and residents. Consequently, we collected additional data following this event as part of our iterative research process to increase the number of case reviews with patients present.

Once all data had been coded, we focused our analysis on multiple comparisons. Based on our research questions this meant comparing data for between case type (new vs. follow-up) and resident level (junior vs. senior). Because junior vs. senior differences were not as evident as we first assumed, we also defined and then coded for strong and weak case presentations, as the level of learner did not always reflect this aspect. We defined strong and weak case presentations through consensus between SH (a rheumatologist with over 20 years of experience with learners) and AA (a final year [second year] rheumatology resident) based on an overall global assessment of the case. This assessment considered the clarity, coherence, and necessary thoroughness of the resident’s case summary, as well as the degree to which relevant reasoning tasks were explicitly addressed or appropriately inferred. Data collection ended when conceptual depth was achieved through the analysis process [33].

### Research Ethics

The procedures used in this study adhere to the tenets of the Declaration of Helsinki. This study has received ethics approval from Lawson (Hospital Ethics- Research Database Application #521) and Western University’s Research Ethics Board (6822). Informed consent was obtained by all study participants.

## Results

Data consisted of 53 case reviews and their associated transcripts in ambulatory rheumatology clinics. Cases reflected a wide range of clinical presentations in ambulatory rheumatology clinics, including seropositive inflammatory arthritis, systemic rheumatic connective tissue disorders, vasculitis, osteoarthritis, and seronegative spondyloarthropathies. Case reviews recording times ranged from 2 min to 25 min with a mean of approximately 7 minutes. Case reviews involved a total of five attending physicians and 24 residents. Of these, 39 were follow-up cases (29 with junior residents [PGYs 1–3], 10 with senior residents [PGYs 4–5]) and 14 were new consultations (10 with junior residents and 4 with senior residents), with some residents and attendings participating in more than one case. In addition, 23 of the 53 case reviews included interactions where patients were present, offering a more complete picture of the reasoning tasks being addressed. A detailed breakdown of case type and level of trainee is provided in Table 1.

**Table 1:** Summary of Case Reviews.


LEVEL OF TRAINING	NEW CASE	FOLLOW-UP CASE	TOTAL

PGY*–1	4	8	12

PGY-2	5	10	15

PGY-3	1	11	12

PGY-4	3	9	12

PGY-5	1	1	2

**Total**	**14**	**39**	**53**


*PGY: Post-Graduate Year.

All reasoning tasks identified during the case reviews were adequately described in the previously developed template of reasoning tasks. However, one reasoning task that had been previously noted for in-patients was not addressed by the residents in ambulatory clinics; *assess decision-making capacity*. On average, new consultations involved 8 (range: 3–12) distinct reasoning tasks per review, and follow-up involved 7 (range: 1–14) distinct reasoning tasks.

In the sections below we describe the difference in patterns of reasoning tasks used in new consultations versus follow-up cases. Within each of these case types we explore differences in reasoning tasks attended to at the level of the trainee (junior vs. senior resident) and those omitted or added by the attending, as well as what differentiates a strong case presentation from a weak case presentation (Supplementary File 1), as the resident training level did not consistently reflect clinical reasoning ability at the level of an individual case.

### New Consultation Case Reviews

All three of the overarching reasoning tasks were addressed in most new consultations – *identify active issues* (12/14), *determine most likely diagnosis and underlying cause(s)* (11/14) and *establish management plan* (10/14), the latter of which was one of the reasoning tasks most often added or refined by the attending. Supportive subtasks that appeared most frequently, included *consider and prioritize differential diagnosis including most likely diagnosis and most serious diagnosis to rule out* (12/14), *select diagnostic investigations taking into account goals of care* (9/14), and *assess severity* (8/14). *Consider the consequences of management on comorbid illnesses, establish goals of care*, and *explore collaborative roles for patient and family* were not addressed in new cases and were tasks exclusively seen in follow-up cases.

As shown in Table 2, there were no consistent differences noted between the number of tasks addressed by junior and senior residents. The rheumatology experts on the research team felt that the number of reasoning tasks attended to was not a marker of expertise. Rather it was selectivity of tasks addressed and the depth with which the tasks were addressed in the context of specific patient needs. In addition, there were a few reasoning tasks that, when addressed, appeared to distinguish stronger from weaker case presentations for new consultations, including *consider the consequence of management on comorbid illnesses* and its corollary *consider the impact of comorbid illness on management* as well as *consider alternative treatment options*.

**Table 2:** Reasoning Tasks Addressed During Case Reviews.


REASONING TASK^17^	NEW^b^ (JUNIOR) (n^a^ = 10)	NEW (SENIOR) (n = 4)	FOLLOW-UP^c^ (JUNIOR) (n = 29)	FOLLOW-UP (SENIOR) (n = 10)	TOTAL (n = 53)

**A. Identify Active Issues**	9 (90%) (Cases 3, 7, 9, 11, 19, 20, 35, 44, 48)	3 (75%) (Cases 36, 37, 53)	24 (83%) (Cases 1, 2, 5, 8, 12, 13, 14, 21, 22, 24, 25, 26, 29, 31, 32, 33, 34, 39, 40, 41, 42, 43, 45, 51)	8 (80%)_ (Cases 6, 10, 15, 16, 17, 23, 38, 47)	44 (83%)

A1. Assess Priorities	2 (20%) (Cases 7, 11)	1 (25%) (Case 37)	2 (69%) (Case 18, 31)	2 (20%) (Cases 10, 38)	7 (13%)

A2. Reprioritize Based on Assessment	3 (30%) (Cases 7, 9, 11)	0 (0%)	2 (69%) (Cases 13, 14)	0 (0%)	5 (94%)

A3. Consider And Prioritize Diagnosis Including Most Likely Diagnosis and Most Serious Diagnosis to Rule Out	9 (90%) (Cases 3, 7, 9, 11, 20, 35, 44, 48, 52)	3 (75%) (Cases 27, 36, 37)	15 (52%) (Cases 1, 5, 8, 13, 21, 22, 29, 32, 33, 39, 40, 41, 42, 43, 51)	4 (40%) (Cases 6, 38, 46, 47)	31 (59%)

A4. Identify Precipitants or Triggers to The Current Problem(s)	3 (30%) (Cases 19, 35, 44)	1 (25%) (Case 37)	3 (10%) (Cases 13, 33, 40)	0 (0%)	7 (13%)

A5. Select Diagnostic Investigations Taking into Account Goals of Care	6 (60%) (Cases 9, 19, 20, 35, 44, 48)	3 (75%) (Cases 27, 36, 37)	10 (34%) (Cases 13, 21, 22, 31, 33, 40, 41, 42, 43, 51)	4 (40%) (Cases 6, 10, 15, 17)	23 (43%)

**B. Determine Most Likely Diagnosis with Underlying Cause(s)**	8 (80%) (Cases 3, 7, 9, 11, 19, 20, 35, 52)	3 (75%) (Cases 27, 36, 37)	14 (48%) (Cases 13, 14, 18, 21, 22, 31, 32, 33, 34, 39, 40, 41, 43, 50)	4 (40%) (Cases 6, 17, 30, 38)	29 (55%)

B1. Identify Modifiable and Non-Modifiable Risk Factors	7 (70%) (Cases 3, 7, 9, 11, 19, 20, 35)	0 (0%)	3 (10%) (Cases 24, 31, 33)	2 (20%) (Cases 6, 15)	12 (23%)

B2. Identify Complications Associated with Diagnosis, Diagnostic Investigations, or Treatment	6 (60%) (Cases 3, 9, 19, 20, 35, 44)	1 (25%) (Cases 37)	17 (59%) (Cases 1, 5, 8, 13, 14, 24, 31, 32, 33, 34, 39, 40, 41, 42, 43, 49, 51)	4 (40%) (Cases 10, 15, 30, 38)	28 (53%)

B3. Assess Rate of Progression, Response to Treatment and Estimate Prognosis and Length of Stay	4 (40%) (Cases 35, 44, 48, 52)	2 (50%) (Cases 36, 37)	19 (66%) (Cases 1, 2, 8, 13, 14, 24, 26, 28, 29, 32, 33, 34, 39, 40, 41, 42, 43, 45, 50)	7 (70%) (Cases 10, 15, 16, 17, 30, 38, 47)	32 (60%)

B4. Explore Physical and Psychosocial Consequences of Current Medical Conditions or Treatment	6 (60%) (Cases 3, 7, 35, 44, 48, 52)	0 (0%)	7 (24%) (Cases 2, 12, 13, 14, 33, 40, 41)	0 (0%)	13 (25%)

B5. Establish Goals of Care	0 (0%)	0 (0%)	7 (24%) (Cases 13, 14, 18, 21, 32, 34, 50)	0 (0%)	7 (13%)

B6. Explore Interplay of Psychosocial Context and Management	3 (30%) (Cases 3, 35, 44)	2 (50%) (Cases 27, 36)	2 (7%) (Cases 18, 31)	0 (0%)	7 (13%)

B7. Consider Impact of Comorbid Illness on Management	1 (10%) (Case 20)	1 (25%) (Case 27)	4 (14%) (Cases 18, 39, 41, 49)	1 (10%) (Case 10)	7 (13%)

B8. Consider the Consequences of Management on Comorbid Illnesses	0 (0%)	0 (0%)	3 (10%) (Cases 18, 25, 51)	1 (10%) (Case 16)	4 (8%)

B9. Consider Alternative Treatment Options	2 (20%) (Cases 20, 48)	2 (50%) (Cases 27, 53)	9 (31%) (Cases 2, 12, 13, 18, 21, 25, 32, 34, 49)	1 (10%) (Case 16)	14 (26%)

B10. Consider Implications of Available Resources on Diagnostic or Management Choices	1 (10%) (Case 52)	0 (0%)	1 (3%) (Case 29)	1 (10%) (Case 38)	3 (6%)

**C. Establish Management Plan**	8 (80%) (Cases 3, 7, 9, 11, 35, 44, 48, 52)	2 (50%) (Cases 36, 37)	24 (83%) (Cases 1, 2, 4, 8, 12, 13, 14, 21, 24, 25, 26, 28, 29, 31, 32, 33, 34, 39, 40, 41, 42, 43, 49, 50)	9 (90%) (Cases 6, 10, 15, 16, 17, 23, 30, 38, 46)	43 (81%)

C1. Select Education and Counselling Approach for Patient and Family	1 (10%) (Case 52)	1 (25%) (Case 53)	7 (24%) (Cases 2, 12, 13, 14, 21, 24, 50)	1 (10%) (Case 47)	10 (19%)

C2. Explore Collaborative Roles for Patient and Family	0 (0%)	0 (0%)	1 (3%) (Case 34)	0 (0%)	1 (2%)

C3. Determine Follow-Up and Consultation Strategies	3 (30%) (Cases 3, 44, 48)	2 (50%) (Case 27, 36)	12 (41%) (Cases 1, 2, 8, 13, 14, 31, 33, 34, 39, 49, 50, 51)	9 (90%) (Cases 6, 10, 15, 17, 23, 30, 38, 46, 47)	26 (49%)

C4. Determine What to Document and Who Should Receive Documentation	3 (30%) (Cases 7, 44, 52)	1 (10%) (Case 27)	2 (7%) (Cases 50, 51)	1 (10%) (Case 38)	7 (13%)

C5. Assess Severity	7 (70%) (Case 3, 11, 19, 20, 35, 44, 48)	1 (10%) (Case 37)	18 (62%) (Cases 1, 2, 5, 8, 12, 18, 24, 29, 31, 32, 33, 39, 40, 41, 42, 45, 49, 50)	6 (60%) (Cases 10, 17, 23, 30, 38, 47)	32 (60%)

C6. Assess Decision-Making Capacity	0 (0%)	0 (0%)	0 (0%)	0 (0%)	0 (0%)


^a^n = number of case reviews that addressed that specific reasoning task. ^b^New Consultation. ^c^Follow-Up Visit. **Legend:** Table 2 summarizes the results of the template analysis. The “Reasoning Task” column presents the previously developed reasoning tasks [^17], with the three overarching categories shown in **bold** and labeled A–C, followed by their corresponding sub-tasks listed alpha-numerically. The next columns indicate the total number of new and follow-up cases, further divided by junior and senior residents. Case review numbers (1–53) identify which reviews addressed each reasoning task, followed by the total count (n) and percentage (%) of case reviews in which that task appeared.

In the following example, a senior resident has considered the treatment options based on co-morbidities, and in fact lists that fact up front:

“*Attending: What do you want to do for him [rheumatoid arthritis]?*

*Resident: […] He is diabetic, but […] he needs some […] prednisone…*

*Attending: […] What do you want to use for DMARDs because he drinks [alcohol]?*

*Resident: […] Plaquenil and sulfasalazine*.” – Case 27 (New Case, Senior [PGY-4], No Patient Present)

In the above example, the resident is applying content specificity to *consider impact of comorbid illness on management* and *consider alternative treatment options*. Weaker case presenters appeared to not recognize these same subtasks which were commonly refined by the attending physician. For example:

“*Attending: So how would you want to treat [her psoriatic arthritis]?*

*Resident: Methotrexate*.

*Attending: If you look up GRAPPA guidelines […], it breaks [treatment] down into different domains of psoriatic arthritis so peripheral arthritis, axial arthritis, enthesitis, dactylitis, skin psoriasis and nail change. So, the first line would be NSAIDs […] sometimes we inject with steroids under imaging guiding through tendon sheath and then methotrexate. But it’s kind of hard to convince someone to go on methotrexate for one digit unless it’s really bothering her, but it might help her skin psoriasis as well*.” – Case 20 (New Case, Junior [PGY-2], No Patient Present)

*Establish management plan*, when addressed, also signalled expertise, and was commonly added or refined by attendings when the patient was present, highlighting its importance in strong case presentations. In addition to addressing specific reasoning tasks, a clear upfront case synthesis, where the right reasoning tasks and not more were addressed, and content specificity also seemed to signal a stronger presentation would ensue. A strong presentation began with a concise synthesis including key features of clinical history, relevant investigations, and differential and most likely diagnosis. In contrast, weaker presentations would omit relevant data or include extraneous information.

### Follow-Up Case Reviews

Of the three overarching tasks, *establish management plan* (33/39) and *identify active issues* (32/39) were addressed most often. *Determine most likely diagnosis with underlying cause(s)* was less commonly addressed explicitly in follow-up cases by the residents (18/39). Attending physicians were most likely to add this overarching task, suggesting that even in follow-up cases it could be important but less likely to be recognized by residents. The most common and, seemingly important – these were the ones also more commonly added or refined by the attending physician – subtasks were *assess rate of progression, response to treatment and estimate prognosis and length of stay* (26/39), *assess severity* (26/39), *identify complications associated with diagnosis, diagnostic investigations, or treatment* (21/39) and *determine follow-up and consultation strategies* (21/39).

Like new cases, the number of reasoning tasks attended to did not predict the strength of the follow-up case presentations. There were several reasoning tasks that, when addressed, appeared to distinguish stronger from weaker case presentations including *identify complications associated with diagnosis, diagnostic investigations, or treatment*, and *assess rate of progression, response to treatment and estimate prognosis and length of stay*.^1^ *Determine follow-up and consultation*, when addressed, also signalled expertise, and was commonly added or refined by attendings when the patient was present, highlighting its importance in strong case presentations.

Again, the selectivity, content specificity, and depth with which the tasks were addressed also helped distinguish stronger and weaker presentations. For example, a strong presentation might only include five reasoning tasks addressed to the right level of detail, and a weaker one could include more tasks addressed superficially.

The following is an example of a strong case presentation.

“*[Raynaud’s] is pretty controlled, and she hasn’t had ulcers since 9 months ago…She has [some] calcinosis […] of her digits right now […] with regards to GERD, she is on ranitidine, domperidone, esomeprazole, and still wakes up in the night twice with […][GERD] and takes Tums […] she hasn’t had any […] cough or shortness of breath and there are no crackles on exam […] Her RVSP was 25–30*.” – Case 31 (Follow-Up, Junior [PGY-2], No Patient Present)

Here, the resident addressed *assess rate of progression, response to treatment and estimate prognosis and length of stay* in a patient with scleroderma with the appropriate level of depth.

In addition, the ability to present a clear, upfront case synthesis, seemed to signal that a stronger presentation would ensue. For example, an excellent presentation would begin with a concise synthesis that included all the key features of the history, physical, investigations, prior treatments and responses to treatment. In complex cases, it would also include previous specialists seen and impressions. The data provided would only include pertinent – to this visit – details like the example below.

“*Mrs. X is a 63-year-old patient with psoriatic arthritis as well as osteoarthritis of her knees. She was initially diagnosed with psoriatic arthritis back in 2014 and she’s been on the Enbrel and methotrexate pretty well controlled for a while. Most of her [active] symptoms have been with regard to her knee [osteoarthritis] which have kind of been the main things on previous visits*.” – Case 38 (Follow-Up, Senior [PGY-4], No Patient Present)

Weaker presentations, on the other hand, would either omit relevant data or include extraneous details.

“*Mrs. X. She is a lady with psoriatic arthritis and osteoarthritis with multiple [joint] replacements. Twice in the hip. Left knee. She is currently waiting for a right shoulder replacement, but you’ve seen her about a month ago and started her on methotrexate and Sulfasalazine and she’s on a small dose of prednisone for psoriatic arthritis. The reason she’s here a bit early is because her right knee is flaring up. Seems like she wants an injection*.” – Case 34 (Follow-Up, Junior [PGY-3], Patient Present)

These distinctions in the clarity and relevance of case synthesis directly influence the overall effectiveness of clinical presentations, often determining the difference between a strong and weak case presentation.

## Discussion

This study explored reasoning tasks in an ambulatory rheumatology setting and examined how the tasks relate to clinical expertise across different case types and training levels. In our discussion, we highlight potential implications for assessment, feedback and future research.

While there have been attempts to use checklists of reasoning tasks for purposes of assessment [34], our findings would argue against this approach; all reasoning tasks should not be addressed in every patient encounter. Regardless of whether the encounter was new or a follow-up, it was not the number of tasks addressed that mattered most, but rather the careful selection of tasks and the extent to which their depth and specificity were tailored to the patient’s particular needs. This finding suggests that, from a cognitive perspective, the reasoning tasks are likely content and context specific and therefore vary based on key patient features [35].

Although the number of reasoning tasks attended to may not signal expertise, certain tasks were associated with strong case presentations. For example, the ability to consider co-morbid illness in management demonstrates a more nuanced approach. These patterns, related to reasoning tasks that were more likely to be addressed by residents in stronger presentations, or added in or refined by the attending for weaker ones. Prior research in clinical reasoning has shown similar expertise effects. For example, research on the development of illness scripts and instance scripts demonstrates that over time, through the process of encapsulation – the process by which detailed biomedical knowledge becomes condensed into higher-level clinical concepts through repeated clinical experience – trainees develop detailed information about consequences and management of specific clinical presentations [6,13]. What this literature and its uptake into feedback and assessment have not, however, achieved are sufficient granularity around aspects of script formation or application with which trainees may be struggling.

This study adds a more nuanced understanding of aspects of the developmental pathways through which trainees learn to reason through clinical problems as well as a clearer view of the specific reasoning tasks that may remain under-addressed. One of the greatest complaints from both trainees and program directors is the lack of specificity around feedback arising during formative, workplace-based assessments [26,36]. Our findings provide a language for changing this. While there are many instances of faculty probing resident thinking around reasoning tasks that were missed or incompletely addressed, such as assessing severity, determining differential and most likely diagnosis and considering alternative treatment options, in none of these instances did faculty explicitly reference the metacognitive elements of the reasoning tasks they were probing or refining. As has been suggested in other research [37,38], doing so can be invaluable in supporting trainee reasoning. For example, instead of stating that a resident’s case review was not strong or was incomplete (ineffective feedback), faculty could clearly identify gaps in the reasoning tasks addressed (e.g., the resident did not consider the effects of comorbidities on management decisions, or the resident failed to explore the psychosocial implications of current medical conditions). Similarly, the commonly missed tasks, that are only addressed in stronger presentations, could serve as the basis for further developing competency-based assessment tools. Developing such tools would be akin to the key-features approach used for other forms of testing where, rather than focusing on generic thoroughness, attention is given instead to the more challenging and discriminating features [39].

Some of the reasoning tasks such as *explore physical and psychosocial consequences of current medical conditions or treatment, select education and counselling approach for patient and family*, and *determine what to document and who should receive documentation* were only discussed in a small sub-set of cases and, as focus group participants pointed out, mostly in the senior resident longitudinal clinic. While the first two of these tasks were infrequently addressed by junior residents in our study, they are regarded as important tasks for trainees to learn and can be challenging to teach [40,41]. By explicitly labelling them, we are able to flag this teaching gap and would suggest that they offer another important element that can be incorporated into teaching in the clinic.

Our findings also have methodological implications. Building on prior work in inpatient settings [10], we initially did not include case reviews occurring in the patient room, which may have led to missing key reasoning tasks tied to each encounter. Future studies should incorporate both types of case review conversations, those just between the attending and the resident, and those conversations extended into the room with the patient. Additionally, many tasks, such as those related to documentation, are reasoned about by attendings but not explicitly discussed. The selectivity in which tasks are refined likely depends on factors like clinic time constraints [11], the level of care (single-visit vs. longitudinal), and the need to avoid overwhelming trainees. Some tasks may also be addressed during charting or post-clinic debriefs [42,43]. Exploring these and self-reflective tasks from the original list of reasoning tasks [8] would require a different approach to data collection.

This study has a few key limitations. The most significant is the transferability of the specific reasoning tasks deemed most important. While we selected the Rheumatology clinic as a typical ambulatory specialty clinic, the findings may not transfer to other clinic types or specialties beyond internal medicine. To mitigate this limitation, we have provided a rich description of the practice context in which the observations took place, enabling readers to assess the relevance and transferability of these findings to their own clinical settings. Future research into other clinical types and other specialities is warranted. Additionally, though we inferred when reasoning tasks were being addressed and only coded them when evident, these inferences may not be accurate; a limitation noted by others in the literature [15]. To address this limitation, we employed analyst triangulation, whereby multiple researchers independently reviewed the transcripts and then worked collaboratively to reach consensus on the reasoning tasks inferred. Another limitation of this study relates to the subjectivity inherent in our categorization of “strong” and “weak” case presentations. These definitions were informed by consensus among the research team, including experienced rheumatology educators. However, we acknowledge that such categorizations are inherently interpretive and shaped by the clinical and pedagogical perspectives of the rheumatology experts involved, limiting their transferability. Lastly, clinical reasoning is a continuous process, and our study may have missed parts of it, especially those communicated implicitly. This could result in an incomplete picture of the reasoning tasks enacted, potentially underrepresenting tasks that are less explicitly articulated or more intuitive in nature. Consequently, our findings may skew toward those reasoning tasks that are more easily observed and may not fully reflect the depth or complexity of the residents’ clinical reasoning.

Our study highlights the importance of specificity and depth in reasoning tasks over quantity. Future work should explore whether and how recognizing and explicitly labeling key metacognitive tasks – through a shared language around clinical reasoning – can enhance trainee development, facilitate more meaningful feedback, and inform the design of competency-based assessments. Such an approach could help residents see beyond the single case, fostering transferable reasoning skills that support expertise development and ultimately improve patient care.

## Data Accessibility Statement

The data supporting this study’s findings are not publicly available due to participant consent restrictions. De-identified data are securely stored at Western University with access limited to the research team.

## Additional File

The additional file for this article can be found as follows:

10.5334/pme.2294.s1 Supplementary File 1. Examples of Strong and Weak Case Presentations.

## Footnotes

Length of stay is not relevant in ambulatory setting and estimating prognosis often refers to a long-term prognosis over months to years– so there are some differences in how this reasoning task is attended to in ambulatory settings, attending to these differences can signal a strong case presentation.
