# Supplementary material for: In Support of Meaningful Assessment and Feedback: A Study of Clinical Reasoning Tasks Used in Ambulatory Case Reviews
Source: Perspect Med Educ. 2026 Mar 13;15(1):257–69. doi: 10.5334/pme.2294 (PMC12985855; doi:10.5334/pme.2294)
Supplement: Supplementary File 1. — Examples of Strong and Weak Case Presentations. [file pme-15-1-2294-s1.pdf]

Supplementary File 1: Examples of Strong and Weak Case Presentations

Article Title: In Support of Meaningful Assessment and Feedback: A Study of Clinical Reasoning Tasks Used in Ambulatory Case Reviews
Journal Name: Perspectives on Medical Education
Author Names: Jacqueline M. I. Torti, Susan Humphrey Murto, Kristen A. Bishop, Azin Ahrari, Mark Goldszmidt
Affiliation and E-Mail Address of the Corresponding Author: Dr. Jacqueline Torti, Centre for Education Research and Innovation, Schulich School of Medicine and Dentistry, Western University. Medical Sciences Building Suite 102A, London, Ontario, Canada N6A 5C1. Email: jtorti2@uwo.ca

**Strong Presentation: Concise Summary Followed by Relevant and Content Specific Information**
"82-year-old man referred to us with inflammatory arthritis. He is retired [..]. Past med history: he's had a previous stroke, hypertension, dyslipidemia, diabetes, urinary retention […] and right hip replacement after a fall and a fracture a few years ago. […] He's on tramadol and acetaminophen for pain and then he's on Plavix - he doesn't take any NSAIDs with that, Amlodipine, Januvia, Lipitor. He's a non-smoker. He's a pretty […] heavy drinker. He has twelve beers a week. […] He said he started to notice swelling of both wrists, multiple MCP joints and he's got what looks like a tenosynovitis at the wrist, like RA kind of looking […] it has only been the past few months that he's got swelling in both wrists, MCP's and PIP's and he said that it has actually progressed. He has such pronounced swelling that he's getting numbness and tingling in all his fingers, so I am wondering if he is just getting carpal tunnel from all the swelling. […] Review of systems is otherwise non-contributory for seronegative diseases. His low back pain doesn't sound inflammatory. And no constitutional symptoms. So, in terms of investigations, he's RF and ANA negative. Inflammatory markers were quite elevated so ESR 58 CRP of 18 in September." – Case 27 (New Case, Senior [PGY-4], No Patient Present)
**Weak Presentation: Contains Extraneous Information and Omits Relevant Information**
"This is Ms. X. A 66-year-old female referred to us for consideration of whether she has inflammatory arthritis and also assistance with osteoarthritis because they are having a lot of difficulties with pain treatments so far. She is retired. She did work at a factory and there she did a lot of repetitive maneuvers, a lot of pulleys that she had to pull on. She got some tears in her rotator cuff that led to a right shoulder arthroplasty in addition to some osteoarthritis in the underlying joint from all those repetitive motions as well. Other than that, her past medical history is she had a cerebral aneurism in 2002. She had that coiled and she has some aphasia, so her daughter is with her and assists with questions. If the question is too complicated, she has trouble answering. She also has hypothyroidism, depression, dyslipidemia. She did have a diagnosis of IBS but the daughter said more recently they were thinking it was just diarrhea from a medication so that may not necessarily be a diagnosis for her anymore. She has had her right shoulder replaced like we spoke about, as well as the left knee. The left knee was replaced for osteoarthritis. […] She has symptoms on bilateral shoulders, bilateral knees. Left shoulder worse

than the right shoulder—so the one without the arthroplasty. And then right and left knees are both hurting her including the one that she had the joint replacement […] she's on tramacet […] started last week […] it's actually working really well [...] She is on [antihypertensive]." – Case 48 (New Case, Junior [PGY-1], Patient Present)

Abbreviations: MCP=metacarpal phalangeal joint; ESR=erythrocyte sedimentation eate; CRP=C-reactive protein; RF=rheumatoid factor; NSAID=non-steroidal anti-inflammatory drug; ANA=antinuclear antibodies

Descriptive Caption: Examples illustrating characteristics of strong versus weak case presentations in postgraduate medical education. The strong presentation demonstrates a concise, structured summary focused on relevant, content-specific clinical details, while the weak presentation contains extraneous background information, omits pertinent findings, and lacks a clear, prioritized organization.
